# Molecular diagnosis of *Plasmodium ovale* by photo-induced electron transfer fluorogenic primers: PET-PCR

**DOI:** 10.1371/journal.pone.0179178

**Published:** 2017-06-22

**Authors:** David Akerele, Dragan Ljolje, Eldin Talundzic, Venkatachalam Udhayakumar, Naomi W. Lucchi

**Affiliations:** 1 Division of Pediatric Infectious Diseases, Emory Medical Center, Atlanta, Georgia, United States of America; 2 Atlanta Research and Education Foundation, Decatur, Georgia, United States of America; 3 Malaria Branch, Division of Parasitic Diseases and Malaria, Center for Global Health, Centers for Disease Control and Prevention, Atlanta, Georgia, United States of America; Université Pierre et Marie Curie, FRANCE

## Abstract

Accurate diagnosis of malaria infections continues to be challenging and elusive, especially in the detection of submicroscopic infections. Developing new malaria diagnostic tools that are sensitive enough to detect low-level infections, user friendly, cost effective and capable of performing large scale diagnosis, remains critical. We have designed novel self-quenching photo-induced electron transfer (PET) fluorogenic primers for the detection of *P*. *ovale* by real-time PCR. In our study, a total of 173 clinical samples, consisting of different malaria species, were utilized to test this novel PET-PCR primer. The sensitivity and specificity were calculated using nested-PCR as the reference test. The novel primer set demonstrated a sensitivity of 97.5% and a specificity of 99.2% (95% CI 85.2–99.8% and 95.2–99.9% respectively). Furthermore, the limit of detection for *P*. *ovale* was found to be 1 parasite/μl. The PET-PCR assay is a new molecular diagnostic tool with comparable performance to other commonly used PCR methods. It is relatively easy to perform, and amiable to large scale malaria surveillance studies and malaria control and elimination programs. Further field validation of this novel primer will be helpful to ascertain the utility for large scale malaria screening programs.

## Introduction

Malaria is caused by protozoan parasites of the genus *Plasmodium* that infect humans through the bite of an infective female A*nopheles* mosquito. There are five different species of *Plasmodium* parasites that commonly cause human disease: *P*. *falciparum*, *P*. *malaria*, *P*. *ovale*, *P*. *vivax*, and *P*. *knowlesi* (zoonotic). *P*. *falciparum* is the most lethal human malaria parasite and is the most prevalent in sub-Saharan Africa [1]. *P*. *vivax* is the most widely distributed geographically and best adapted to survive in temperate climates.

Both *P*. *ovale* and *P*. *malariae* mainly occur in certain areas of sub-Saharan Africa, with *P*. *ovale* occurring mainly in West Africa. *P*. *ovale* was one of the last human malaria parasites to be described. The natural distribution of *P*. *ovale* was initially thought to be restricted to sub-Saharan Africa and the islands of the western Pacific [2, 3], but recent studies have noted *P*. *ovale* to also circulate in India, Bangladesh, Vietnam, and Myanmar [4–7] Malaria caused by *P*. *ovale* infection has been considered a low-prevalence disease with limited geographic distribution, benign clinical course, and easy treatment; therefore, little attention has been paid to it [8] Diagnosis of *P*. *ovale* has usually been made through microscopy. However, this can be challenging due to the fact that many *P*. *ovale* infections present with low levels of parasitemia, and therefore, highly sensitive tools may be required to detect it. In addition, mixed infections with other *Plasmodium* species, especially *P*. *falciparum*, might compromise the detection of *P*. *ovale*. There are no rapid diagnostic tests (RDTs) specific for *P*. *ovale*; detection of non-falciparum Plasmodium infections by RDT is limited to the use of a Pan (all Plasmodium) test. This does not allow for the discrimination of the non-falciparum infections.

Interestingly, this neglected *Plasmodium* species has two distinct subspecies that are essentially morphologically and clinically indistinguishable, but are separated by subtle genetic dimorphisms [9, 10]: *P*. *ovale curtisi* (classic form) and *P*. *ovale wallikeri* (variant form) [11]. These two subspecies circulate simultaneously in sub-Saharan Africa [11] and in Asia [4–7]and therefore, it is important to have a clinical diagnostic test that can accurately detect both of these subspecies.

Based on the recent success in global efforts to reduce the number of malaria cases and deaths [1], there is momentum to control and eliminate malaria. In this vain, there is a clear need for accurate diagnostic tools to detect the species of infecting parasites, to identify the transmission foci and malaria reservoirs (which may comprise asymptomatic patients with submicroscopic infections) and to monitor the success of malaria control and elimination programs.

Malaria elimination and control programs utilize three major diagnostic tools: antigen/antibody based RDTs, microscopy, and molecular tools (nucleic acid based tools). Microscopy remains the gold standard for diagnosis of malaria in many malaria-endemic countries. While microscopy is inexpensive, can quantify parasite burden, and can differentiate parasite species, it has several limitations. Preparation of blood smears is laborious, difficult to standardize, and the diagnosis of low parasite density is challenging and requires seasoned microscopists. Inter-user variability (a two to three-fold discrepancy) can occur in parasite quantification especially when routine training and quality management procedures are not practiced [12].

RDTs also have a role in case management and control programs. Some RDTs are *Plasmodium*–specific (pan), detecting the genus-specific aldolase and lactate dehydrogenase enzymes. The vast majority of available RDTs (90%) are specific for *P*. *falciparum* histidine-rich protein 2 (Pf HRP-2), and are therefore limited as they cannot detect other human *Plasmodium* species. Additionally most of these RDTs have a relatively high threshold for detecting *P*. *ovale*, around 100 parasites/μl [13, 14]. Lastly, RDTs are also limited in that they cannot quantify and delineate parasite densities.

Given the limitations of RDTs and microscopy there is a clear need for sensitive, cost effective, and user friendly tools that can complement the current malaria diagnostics. Molecular diagnostic tools are far more sensitive for detecting malaria infections with low parasite burden while offering accurate *Plasmodium* speciation [15, 16]. These tools include, conventional PCR-based assays, real-time PCR assays, and isothermal amplification assays [17].

The real-time PCR platform is advantageous for large scale screening and recently, we have shown that the photo-induced electron transfer (PET)-PCR is a convenient tool for limited resource settings due the ease of use [18, 19]. This PET-PCR assay does not require internal dual-labelled probes or non-specific intercalating dyes. Previously, we described a genus-specific and *P*. *falciparum* multiplex PET-PCR assay [18, 19]. In this study we aimed to design sensitive and specific PET-PCR primers for the detection of both subspecies of *P*. *ovale* in one assay. We evaluated the utility of this assay using 173 clinical samples of different malaria species infections, and using well quantified *P*. *ovale* samples to estimate the limits of detection.

## Methods and materials

### Ethics statement

Clinical samples used in this study were anonymized samples obtained from malaria specimens routinely submitted to CDC reference diagnostic laboratory in the Division of Parasitic Diseases and Malaria for malaria diagnosis. The submission of laboratory specimens are deemed a routine surveillance activity and not a human subjects’ research activity by the CDC IRB. The authors did not have access to any identifying patient information. No human tissues were used in this study. The non-falciparum specimens (*P*. *vivax*, *P*. *malariae and P*. *ovale*) were obtained from CDC’s collections previously obtained from a contractor who used American Association for the Accreditation and Assessment of Laboratory Animal Care (AAALAC) approved protocol for collection of these specimens from non-human primates (chimpanzee or monkeys).

### PET-PCR primers

**Fig 1:**
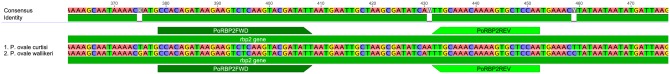
*P*. *ovale* reticulocyte binding protein 2 (*rbp-2*) sequence alignment. Both *P*. *ovale curtisi* and *P*. *ovale wallikeri* sequences were aligned using Geneious software program in order to select a conserved region for the two *P*. *ovale* subspecies. Cytosine is labelled purple, adenine pink, guanine yellow and thymine green. The forward (PoRBP2FWD) and reverse (PoRBP2REV) primers are denoted in dark and light green boxes, respectively.

In this study, *P*. *ovale* specific primers were adapted to the PET-PCR platform based on Miller et al [20]. These primers target the *P*. *ovale* reticulocyte binding protein 2 (*rbp2) gene*. In silico testing, using Geneious 9.1.4 software (http://www.geneious.com) was performed first before primers were synthesized. A BLAST search was performed to ascertain that our selected region of interest on the *RBP-2 gene* was specific to both *P*. *ovale curtisi and P*. *ovale wallikeri* (Fig 1).

In addition to designing *P*. *ovale* primers, we also designed an internal control primer set based on the human *RNase-P gene*. We adapted the *RNase-P gene* sequence described by Luo et al [21]. In both cases, the 5’ end of the forward primers were modified with the PET tag and labeled with FAM (*P*. *ovale*) and HEX (*RNase-P gene*) fluorophores. The PET tag sequence and all other oligonucleotide primers for *P*. *ovale* and *RNase-P gene* are shown in Table 1.

**Table 1:** Oligonucleotide primer sequences used in this study.

Names of Primer	Direction	Sequence (5’-3’)	T_m_ (°C)
PoRBP2FWD	Forward	FAM-agg cgc ata gcg cctggCCACAGATAAGAAGTCTCAAGTACGATATT	60.9
PoRBP2REV	Reverse	TTGGAGCACTTTTGTTTGCAA	57.7
RNase-PFWD	Forward	HEX-agg cgc ata gcg cct ggAGATTTGGACCTGCGAGC	57.3
RNase-PREV	Reverse	GAGCGGCTGTCTCCACA	57.8

The PET tag (lower case: agg cgc ata gcg cct gg) was added to the 5’ end of the *P*. *ovale* and the *RNase-P* forward primers.

### *Plasmodium* parasites and clinical samples

Different *Plasmodium* species acquired from archived whole blood or parasites culture (for *P*. *falciparum* strains) at the CDC were used in this study: 2 strains of *P*. *falciparum* (Dd2, Hb3), 1 *P*. *vivax* (Sal I), 1 *P*. *malariae* (CDC Uganda I), 4 strains of *P*. *knowlesi* (Malayan, Philippines, Nuri, Hackeri), and separate blood samples of *P*. *ovale* (CDC Nigeria I strain) obtained from three different chimpanzees. The non-falciparum specimens were obtained from CDC’s collections that were obtained from a contractor who used American Association for the Accreditation and Assessment of Laboratory Animal Care (AAALAC) approved protocol for collection of these specimens from Chimpanzee or monkeys. In addition, well characterized *P*. *ovale curtisi* and *P*. *ovale wallikeri* DNA samples were kindly provided by Dr. Colin Sutherland’s lab at the London School of Hygiene and Tropical Medicine.

A total of 173 anonymized clinical whole blood samples were obtained from the CDC molecular diagnostic parasitology reference laboratory (40 non-malaria samples, 41 *P*. *falciparum*, 36 *P*. *vivax*, 14 *P*. *malariae*, 40 *P*. *ovale* and 2 unidentified *Plasmodium spp*).

### DNA extraction

DNA was isolated from all the samples using the commercially available QIAamp DNA Mini Kit (QIAGEN, Valencia, CA, USA). The DNA was aliquoted and stored at -20°C until used in the experiments.

### PET-PCR method

All clinical samples were initially tested using a multiplex *Plasmodium* genus (FAM-labeled)/ human RNase-P (HEX-labeled) assay. This initial step detected the presence or absence of *Plasmodium* DNA in each sample and confirmed the successful DNA isolation from the samples. All the samples were subsequently tested using the singleplex *P*. *ovale* assay (FAM -labeled). This last step detected the presence or absence of both subspecies of *P*. *ovale*.

All the PET-PCR assays were performed in a 20 μl reaction mix containing 2X TaqMan Environmental Master Mix 2.0 (Applied BioSystems), 250 nM of each forward and reverse primer, and 5 μl of DNA template. The reactions were performed under the following cycling parameters: initial hot-start at 95°C for 15 minutes, followed by 45 cycles of denaturation at 95°C for 10 seconds, annealing at 62°C (for *P*. *ovale*) and 60°C (*Plasmodium*/*RNase-P*).

The correct fluorescence channel was selected for each primer set and the cycle threshold (CT) values recorded at the end of annealing step. A cut-off CT value of 40.0 or below was used to indicate a positive result.

### Test for specificity

*P*. *falciparum*, *P*. *vivax*, *P*. *malariae*, *P*. *ovale curtisi*, *P*. *ovale wallikeri* and *P*. *knowlesi* samples were utilized for *P*. *ovale* primer specificity testing. The specificity of the *P*. *ovale* primers was evaluated by their ability to only amplify *P*. *ovale curtisi* and *P*. *ovale wallikeri*.

### Analytical sensitivity

To determine the limits of detection of the *P*. *ovale* PET-PCR assay, two-fold serial dilutions of three different blood samples of *P*. *ovale* (CDC Nigeria I strain) obtained from three different chimps were prepared using malaria negative whole blood starting from a parasite density of 2000 parasites/μl to 0.98 parasites/μl. The DNA was then extracted from each dilution, aliquoted, and stored until used in the experiments.

### Clinical sensitivity and specificity

The clinical sensitivity and specificity for the *P*. *ovale* PET-PCR assay was determined using 173 previously diagnosed clinical samples. The study primary investigator was blinded during experiments with these clinical samples. Samples were labelled 1–173, and only the supervising investigator was privy to master-key.

For calculating sensitivity and specificity, the following equations were utilized: Sensitivity = # of true positives / (# of true positives + # of false negatives). Specificity = # of true negatives / (# of true negatives + # of false positives).

## Results

### Primer design

A total of sixteen primer sets were initially designed from selected target candidates: twelve novel primers were designed based on the 18s ribosomal RNA gene (SSU) and four primers were adapted from Miller et al [20], based on the tryptophan-rich antigen (*tra*) gene, and the reticulocyte binding protein 2 (*rbp2*) gene.

Each primer set was first tested with all known human infecting *Plasmodium* species. Primers pairs that correctly amplified *P*. *ovale* and no other species were then evaluated for their ability to amplify both the subspecies of *P*. *ovale*. Using these criteria, one primer set based on the *rbp2 gene* (Fig 1) was selected for further evaluation. The others primers did not meet the set criteria, and were not validated further. This *rbp2*-based primers were then evaluated for their analytical sensitivity using 3 well quantified *P*. *ovale* specimens and for their sensitivity and specificity using clinical samples.

### Limits of detection of PET-PCR assay

Using a Ct value of 40 as the cut off, the *P*. *ovale* PET-PCR assay detected as low as 0.98 parasite per μl (1 parasite per μl) for all the three different *P*. *ovale* preparations with a mean Ct values of 39.47, 39.98, and 39.45.

### Specificity for PET-PCR assay

**Fig 2:**
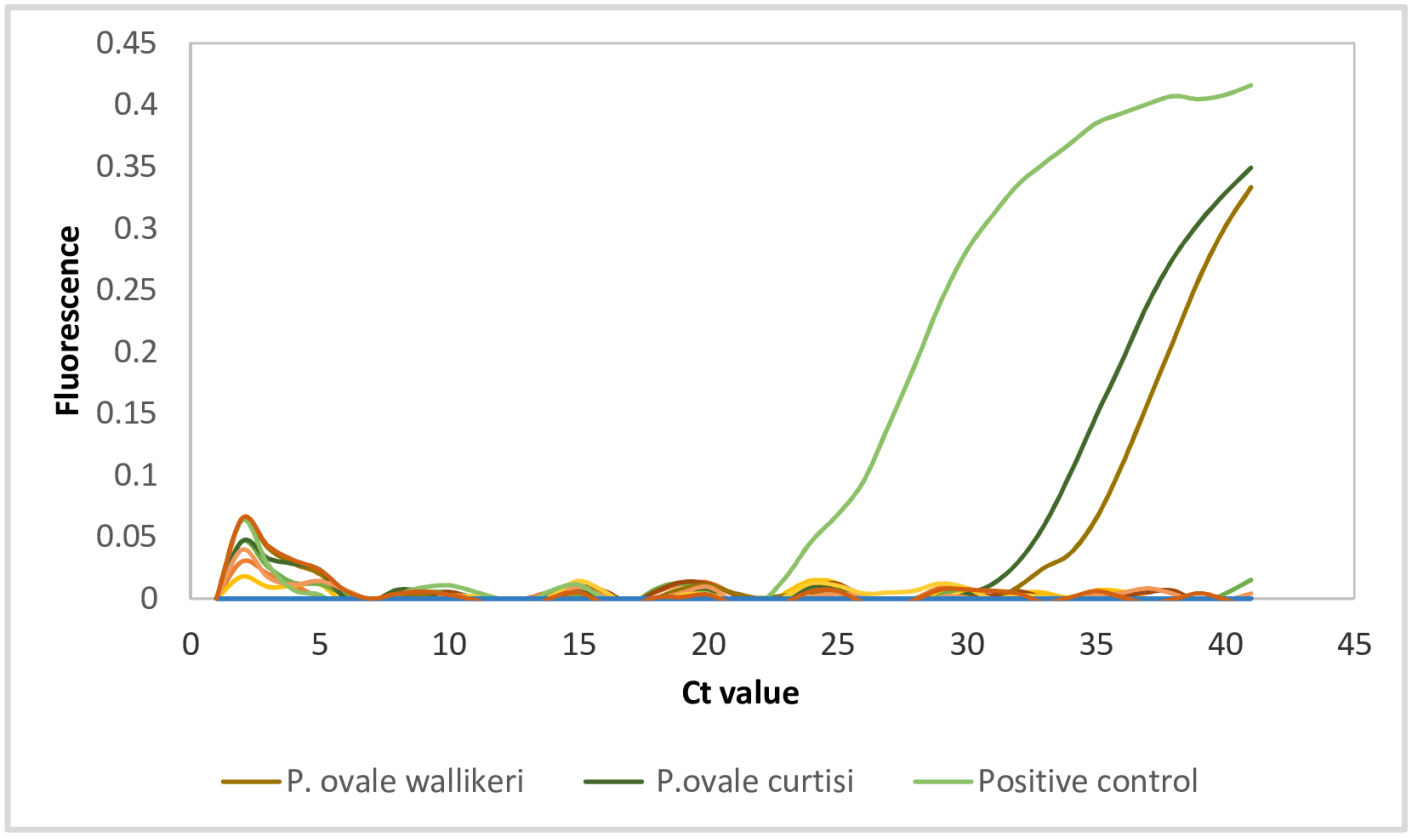
Novel *P*. *ovale* primers only amplify *P*. *ovale* and not the other human-infecting species. *P*. *falciparum*, *P*. *vivax*, *P*. *malariae*, *P*. *knowlesi*, *P*. *ovale curtisi*, *P*. *ovale wallikeri* DNA samples were utilized for primer specificity testing. Only the positive control (a known *P*. *ovale* sample), *P*. *ovale curtisi* and *P*. *ovale wallikeri* were amplified using our primers (amplification plots with Ct values of 25.57, 33.38 and 35.2 respectively). No amplification (flat lines) was noted for the other species and the no template control (NTC).

Our goal was to develop primers that could successfully detected both *P*. *ovale curtisi* and *P*. *ovale wallikeri*, without amplifying any other human *Plasmodium* species. *P*. *falciparum*, *P*. *vivax*, *P*. *malariae*, *P*. *knowlesi*, *P*. *ovale curtisi*, *and P*. *ovale wallikeri* were utilized for primer specificity testing. Only the positive control (a known *P*. *ovale* sample), *P*. *ovale curtisi*, *and P*. *ovale wallikeri* were amplified (Ct values of 25.57, 33.38 and 35.2 respectively), Fig 2. No amplification was noted with *P*. *falciparum*, *P*. *vivax*, *P*. *malariae*, *P*. *knowlesi*, and the negative control (no template control).

### Clinical sensitivity and specificity of the *P*. *ovale* PET-PCR assay

A total of 173 clinical samples, consisting of different malaria species (no mixed infections) and non-malaria samples, were used to test the clinical sensitivity and specificity of the *P*. *ovale* PET-PCR primers. Of the 173 clinical samples tested, the assay identified 39 of the 40 *P*. *ovale* samples. The novel *P*. *ovale* assay identified an additional *P*. *ovale* (Ct of 34.15) sample that was identified as a *P*. *falciparum* by our reference tests. The remaining non- *P*. *ovale* clinical samples did not amplify using our *P*. *ovale* specific primers. The sensitivity and specificity of our assay was calculated using a combination of a real-time PCR and nested-PCR as a reference test. Table 2, shows the calculated sensitivity and specificity to be 97.5% and 99.2% (95% CI 85.2–99.8% and 95.2–99.9% respectively).

**Table 2:** The calculated sensitivity and specificity, of the *P*. *ovale* PET-PCR assay.

PET-PCR	Reference PCR	
	Positive	Negative
**Positive**	39	1
**Negative**	1	132
**Total**	40	133

The sensitivity was calculated as the # of true positives / (# of true positives + # of false negatives). The specificity was calculated as the # of true negatives / (# of true negatives + # of false positives). Sensitivity was calculated to be 97.5% (95% CI 85.2–99.8%) and the specificity was calculated to be 99.2%. (95% CI 95.2–99.9%).

## Discussion

*P*. *ovale* malaria does not get as much attention as other species of *Plasmodium* that cause malaria in humans because of lower morbidity and mortality associated with it. Indeed, it is considered by some to be “the benign malaria,” with less complicated course (4). However there have been many well documented case reports of severe complications associated with *P*. *ovale* such as acute respiratory distress syndrome (ARDS), acute renal failure (ARF) and splenic infarction [22–24]. Globalization, international travel, and migration have increased the incidence of imported malaria in industrialized countries. For example, *P*. *ovale* infections represent up to 8% of imported malaria cases in Italy (mainly from West Africa) [25]. There have been cases of *P*. *ovale* infections imported to the US, Spain, Singapore, and Malaysia from endemic regions [26–29].

*P*. *ovale* infections often time have low parasite burdens [28]. Therefore diagnostic tools for *P*. *ovale* need to be able to detect low parasite densities. In our study, we successfully designed PET-PCR primers for the detection of both subspecies of *P*. *ovale* with good clinical sensitivity (97.5%) and specificity (99.2%). These primers also had good limits of detection, detecting up to 1 parasites per μl. This limit of detection is within the recommended WHO requirements for a nucleic acid tests (2 parasites/μl). The limit of detection of our assay is better than what has been described for microscopy and RDTs [30] being 50 times and 100 times more compared to microscopy and RDTs, respectively. Both microscopy and RDTs are not capable of detecting such low parasite densities and therefore, many *P*. *ovale* infections might go undetected [31]. Our assay identified 1 *P*. *falciparum* sample (by our reference tests) as *P*. *ovale* (Ct of 34.15). It is possible that this is a true *P*. *ovale* sample mixed with *P*. *falciparum* and that our novel primers picked it up correctly or that it was a false positive result. Unfortunately, we were not able to retest this sample as we ran out of DNA from that particular clinical sample. With the success of global malaria elimination initiatives, infections with the more common human malaria parasites (*P*. *falciparum and P*. *vivax*) have decreased significantly [1]. It is therefore plausible to anticipate that infections with the less common parasites (*P*. *ovale* and *P*. *malariae*) may increase. Williams et al, demonstrated changing malaria epidemiology, with increasing *P*. *knowlesi* incidence following the control of *P*. *falciparum* and *P*. *vivax* in Sabah, Malaysia [32]. In regions where *P*. *ovale* circulates, effective malaria control strategies may cause a similar phenomenon leading to increasing *P*. *ovale* infection rates. Therefore, the success of any malaria control program requires that effective diagnostic tools are readily available for the detection of any human *Plasmodium* species, and especially when presented at low parasite densities to allow their accurate detection and prompt treatment. The described *P*. *ovale* PET-PCR assay will contribute towards this program.

There are some limitations to our study. Firstly, we performed retrospective analysis of archived samples available to us from our CDC reference laboratory. It would have been ideal to perform a similar study prospectively. Secondly, the limitation of the achieved samples, all of mono-infections, did not allow us to test the sensitivity and specificity of our primers in mixed infections know to occasionally occur. Finally, we did not further classify the *P*. *ovale* samples identified in this study into *P*. *ovale curtisi* or *P*. *ovale wallikeri* either by sequencing or by using primers specific to these subspecies. However, our primer design was aimed at developing primers capable of detecting the two subspecies in the same assay. We were able to demonstrate that the novel primers we developed detect both *P*. *ovale curtisi* and *P*. *ovale wallikeri* using the known *P*. *ovale curtisi* and *P*. *ovale wallikeri* DNA kindly provided to us by Dr. Colin Sutherland.

In summary, we have designed specific and sensitive PET-PCR primers that are capable of detecting submicroscopic levels of both the subspecies of *P*. *ovale*. It would be helpful to conduct further evaluation in the field to further validate this assay for large scale future field use.

## References

[1] World Health Organization. World Malaria Report. Geneva: World Health Organization, 2015 2015. Report No.

[2] Collins WE, Jeffery GM. Plasmodium ovale: parasite and disease. Clin Microbiol Rev. 2005;18(3):570–81. doi: 10.1128/CMR.18.3.570-581.2005 16020691 10.1128/CMR.18.3.570-581.2005 PMC1195966

[3] Lysenko AJ, Beljaev AE. An analysis of the geographical distribution of Plasmodium ovale. Bull World Health Organ. 1969;40(3):383–94. ; 5306622 PMC2554635

[4] Chaturvedi N, Bhandari S, Bharti PK, Basak SK, Singh MP, Singh N. Sympatric distribution of Plasmodium ovale curtisi and P. ovale wallikeri in India: implication for the diagnosis of malaria and its control. Transactions of the Royal Society of Tropical Medicine and Hygiene. 2015;109(5):352–4. doi: 10.1093/trstmh/trv015 . 25716936 10.1093/trstmh/trv015

[5] Fuehrer HP, Stadler MT, Buczolich K, Bloeschl I, Noedl H. Two techniques for simultaneous identification of Plasmodium ovale curtisi and Plasmodium ovale wallikeri by use of the small-subunit rRNA gene. Journal of clinical microbiology. 2012;50(12):4100–2. doi: 10.1128/JCM.02180-12 ; 23015675 10.1128/JCM.02180-12 PMC3503021

[6] Nguyen HV, van den Eede P, van Overmeir C, Thang ND, Hung le X, D'Alessandro U, et al Marked age-dependent prevalence of symptomatic and patent infections and complexity of distribution of human Plasmodium species in central Vietnam. The American journal of tropical medicine and hygiene. 2012;87(6):989–95. doi: 10.4269/ajtmh.2012.12-0047 ; 23128294 10.4269/ajtmh.2012.12-0047 PMC3516102

[7] Win TT, Lin K, Mizuno S, Zhou M, Liu Q, Ferreira MU, et al Wide distribution of Plasmodium ovale in Myanmar. Trop Med Int Health. 2002;7(3):231–9. . 11903985 10.1046/j.1365-3156.2002.00857.x

[8] Mueller I, Zimmerman PA, Reeder JC. Plasmodium malariae and Plasmodium ovale—the “bashful” malaria parasites. Trends in parasitology. 2007;23(6):278–83. doi: 10.1016/j.pt.2007.04.009 17459775 10.1016/j.pt.2007.04.009 PMC3728836

[9] Calderaro A, Piccolo G, Perandin F, Gorrini C, Peruzzi S, Zuelli C, et al Genetic polymorphisms influence Plasmodium ovale PCR detection accuracy. Journal of clinical microbiology. 2007;45(5):1624–7. doi: 10.1128/JCM.02316-06 ; 17360843 10.1128/JCM.02316-06 PMC1865880

[10] Win TT, Jalloh A, Tantular IS, Tsuboi T, Ferreira MU, Kimura M, et al Molecular analysis of Plasmodium ovale variants. Emerging infectious diseases. 2004;10(7):1235–40. doi: 10.3201/eid1007.030411 ; 15324543 10.3201/eid1007.030411 PMC3323326

[11] Oguike MC, Betson M, Burke M, Nolder D, Stothard JR, Kleinschmidt I, et al Plasmodium ovale curtisi and Plasmodium ovale wallikeri circulate simultaneously in African communities. Int J Parasitol. 2011;41(6):677–83. Epub 2011/02/15. doi: 10.1016/j.ijpara.2011.01.004 ; 21315074 10.1016/j.ijpara.2011.01.004 PMC3084460

[12] O'Meara WP, Barcus M, Wongsrichanalai C, Muth S, Maguire JD, Jordan RG, et al Reader technique as a source of variability in determining malaria parasite density by microscopy. Malaria journal. 2006;5:118 doi: 10.1186/1475-2875-5-118 ; 17164007 10.1186/1475-2875-5-118 PMC1712346

[13] Houze S, Hubert V, Cohen DP, Rivetz B, Le Bras J. Evaluation of the Clearview(R) Malaria pLDH Malaria Rapid Diagnostic Test in a non-endemic setting. Malaria journal. 2011;10:284 doi: 10.1186/1475-2875-10-284 ; 21951996 10.1186/1475-2875-10-284 PMC3196929

[14] Maltha J, Gillet P, Bottieau E, Cnops L, van Esbroeck M, Jacobs J. Evaluation of a rapid diagnostic test (CareStart Malaria HRP-2/pLDH (Pf/pan) Combo Test) for the diagnosis of malaria in a reference setting. Malaria journal. 2010;9:171 doi: 10.1186/1475-2875-9-171 ; 20565816 10.1186/1475-2875-9-171 PMC2906498

[15] Snounou G. Detection and identification of the four malaria parasite species infecting humans by PCR amplification. Methods in molecular biology. 1996;50:263–91. . 8751365 10.1385/0-89603-323-6:263

[16] Johnston SP, Pieniazek NJ, Xayavong MV, Slemenda SB, Wilkins PP, da Silva AJ. PCR as a confirmatory technique for laboratory diagnosis of malaria. Journal of clinical microbiology. 2006;44(3):1087–9. doi: 10.1128/JCM.44.3.1087-1089.2006 16517900 10.1128/JCM.44.3.1087-1089.2006 PMC1393165

[17] Erdman LK, Kain KC. Molecular diagnostic and surveillance tools for global malaria control. Travel medicine and infectious disease. 2008;6(1–2):82–99. Epub 2008/03/18. doi: 10.1016/j.tmaid.2007.10.001 . 18342279 10.1016/j.tmaid.2007.10.001

[18] Lucchi NW, Narayanan J, Karell MA, Xayavong M, Kariuki S, DaSilva AJ, et al Molecular diagnosis of malaria by photo-induced electron transfer fluorogenic primers: PET-PCR. PloS one. 2013;8(2):e56677 doi: 10.1371/journal.pone.0056677 ; 23437209 10.1371/journal.pone.0056677 PMC3577666

[19] Talundzic E, Maganga M, Masanja IM, Peterson DS, Udhayakumar V, Lucchi NW. Field evaluation of the photo-induced electron transfer fluorogenic primers (PET) real-time PCR for the detection of Plasmodium falciparum in Tanzania. Malaria journal. 2014;13:31 doi: 10.1186/1475-2875-13-31 ; 24467985 10.1186/1475-2875-13-31 PMC3917897

[20] Miller RH, Obuya CO, Wanja EW, Ogutu B, Waitumbi J, Luckhart S, et al Characterization of Plasmodium ovale curtisi and P. ovale wallikeri in Western Kenya utilizing a novel species-specific real-time PCR assay. PLoS neglected tropical diseases. 2015;9(1):e0003469 doi: 10.1371/journal.pntd.0003469 ; 25590587 10.1371/journal.pntd.0003469 PMC4295880

[21] Luo W, Yang H, Rathbun K, Pau CP, Ou CY. Detection of human immunodeficiency virus type 1 DNA in dried blood spots by a duplex real-time PCR assay. Journal of clinical microbiology. 2005;43(4):1851–7. doi: 10.1128/JCM.43.4.1851-1857.2005 ; 15815008 10.1128/JCM.43.4.1851-1857.2005 PMC1081318

[22] Lee EY, Maguire JH. Acute pulmonary edema complicating ovale malaria. Clin Infect Dis. 1999;29(3):697–8. . 10530480 10.1086/598667

[23] Lau YL, Lee WC, Tan LH, Kamarulzaman A, Syed Omar SF, Fong MY, et al Acute respiratory distress syndrome and acute renal failure from Plasmodium ovale infection with fatal outcome. Malaria journal. 2013;12:389 doi: 10.1186/1475-2875-12-389 ; 24180319 10.1186/1475-2875-12-389 PMC4228392

[24] Cinquetti G, Banal F, Rondel C, Plancade D, de Saint Roman C, Adriamanantena D, et al Splenic infarction during Plasmodium ovale acute malaria: first case reported. Malaria journal. 2010;9:288 doi: 10.1186/1475-2875-9-288 ; 20955610 10.1186/1475-2875-9-288 PMC2984568

[25] Calderaro A, Gorrini C, Peruzzi S, Piccolo G, Dettori G, Chezzi C. An 8-year survey on the occurrence of imported malaria in a nonendemic area by microscopy and molecular assays. Diagn Microbiol Infect Dis. 2008;61(4):434–9. doi: 10.1016/j.diagmicrobio.2008.03.016 . 18501548 10.1016/j.diagmicrobio.2008.03.016

[26] Chavatte JM, Tan SB, Snounou G, Lin RT. Molecular characterization of misidentified Plasmodium ovale imported cases in Singapore. Malaria journal. 2015;14:454 doi: 10.1186/s12936-015-0985-8 ; 26577930 10.1186/s12936-015-0985-8 PMC4650842

[27] Liew JW, Mahmud R, Tan LH, Lau YL. Diagnosis of an imported Plasmodium ovale wallikeri infection in Malaysia. Malaria journal. 2016;15:8 doi: 10.1186/s12936-015-1070-z ; 26738724 10.1186/s12936-015-1070-z PMC4704402

[28] Rojo-Marcos G, Rubio-Munoz JM, Ramirez-Olivencia G, Garcia-Bujalance S, Elcuaz-Romano R, Diaz-Menendez M, et al Comparison of imported Plasmodium ovale curtisi and P. ovale wallikeri infections among patients in Spain, 2005–2011. Emerging infectious diseases. 2014;20(3):409–16. doi: 10.3201/eid2003.130745 ; 24572501 10.3201/eid2003.130745 PMC3944870

[29] Cohen R, Feghali K, Alemayehu S, Komisar J, Hang J, Weina PJ, et al Use of qPCR and genomic sequencing to diagnose Plasmodium ovale wallikeri malaria in a returned soldier in the setting of a negative rapid diagnostic assay. The American journal of tropical medicine and hygiene. 2013;89(3):501–6. doi: 10.4269/ajtmh.12-0724 ; 23836567 10.4269/ajtmh.12-0724 PMC3771289

[30] Wongsrichanalai C, Barcus MJ, Muth S, Sutamihardja A, Wernsdorfer WH. A review of malaria diagnostic tools: microscopy and rapid diagnostic test (RDT). The American journal of tropical medicine and hygiene. 2007;77(6 Suppl):119–27. . 18165483

[31] Diallo MA, Badiane AS, Diongue K, Deme A, Lucchi NW, Gaye M, et al Non-falciparum malaria in Dakar: a confirmed case of Plasmodium ovale wallikeri infection. Malaria journal. 2016;15(1):429 doi: 10.1186/s12936-016-1485-1 ; 27557982 10.1186/s12936-016-1485-1 PMC4997729

[32] William T, Rahman HA, Jelip J, Ibrahim MY, Menon J, Grigg MJ, et al Increasing incidence of Plasmodium knowlesi malaria following control of P. falciparum and P. vivax Malaria in Sabah, Malaysia. PLoS neglected tropical diseases. 2013;7(1):e2026 doi: 10.1371/journal.pntd.0002026 ; 23359830 10.1371/journal.pntd.0002026 PMC3554533

